# Incidence and management of diarrhoea associated with abemaciclib and endocrine therapy for hormone-receptor positive, HER2-negative metastatic breast cancer: the UK patients’ experiences

**DOI:** 10.1007/s00520-025-09440-7

**Published:** 2025-04-26

**Authors:** Helena Harder, Rachel Starkings, Lesley Fallowfield, Shirley May, Valerie Shilling

**Affiliations:** Sussex Health Outcomes Research and Education in Cancer (SHORE-C), Brighton and Sussex Medical School, University of Sussex, Brighton, UK

**Keywords:** Metastatic breast cancer, Abemaciclib, Side effects, Diarrhoea, Management, Patient experience

## Abstract

**Purpose:**

Addition of a CDK4/6 inhibitor to endocrine therapy (ET) prolongs survival in HR + /HER2-metastatic breast cancer (MBC). Gastrointestinal side effects, predominantly diarrhoea and abdominal pain, are common in patients receiving abemaciclib. This can potentially increase symptom burden, reduce quality of life (QoL) and affect treatment adherence. This longitudinal mixed-methods study with a 6-month follow-up explored patients’ outcomes and experiences.

**Methods:**

Participants (*n* = 44) completed validated QoL measures at study-entry and at 1, 3 and 6 months. Weekly diarrhoea diaries with free-text response options assessed bowel movements and self-management strategies. Optional interviews gathered insight in patients’ experiences.

**Results:**

Forty-two participants completed study measures at study-entry and 24 at 6 months. 17/42 reported no gastrointestinal side-effects. Above threshold diarrhoea (≥ 3 loose/liquid stools daily) was reported at least once by 25/42, with 3/42 having persistent symptoms. Strategies to control diarrhoea, employed by 28/42, included dietary modifications, non-prescribed medication-use and nonadherence (dose interruption or reduction). Meaningful decline on the QoL diarrhoea subscale was observed in 12/37 at 1 month, 13/28 at 3 months and 8/23 at 6 months. Free-text analysis showed that diarrhoea disrupted everyday life in those affected.

**Conclusion:**

A proportion of this small sample of MBC patients treated with abemaciclib and ET-reported diarrhoea which affected symptom burden and QoL. Close symptom monitoring alongside targeted supportive/educational interventions should be introduced to reduce the negative impact on patients’ lives.

**Trial registration:**

ClinicalTrials.gov Identifier: ISRCTN17281696.

**Supplementary Information:**

The online version contains supplementary material available at 10.1007/s00520-025-09440-7.

## Background

The number of people living with incurable metastatic breast cancer (MBC) is expected to increase by more than 50% by 2030 [1]. Although survival has gradually improved over time, the 5-year overall survival rate is 38%, compared to 96% for women with early breast cancer [2].

Treatment for MBC aims to slow further progression and alleviate symptoms and side effects. New targeted therapies have expanded therapeutic options and improved outcomes, particularly for patients with hormone-receptor positive (HR +), human epidermal growth factor receptor 2-negative (HER2-) disease [3]. Current guidelines recommend the addition of a cyclin-dependent kinase 4/6 (CDK4/6) inhibitor, like abemaciclib, in combination with endocrine therapy (ET) based upon multiple clinical trials [4]. MONARCH 2 and 3 evaluated the efficacy and safety of abemaciclib and demonstrated that use of abemaciclib as first-line therapy in combination with an aromatase inhibitor (AI), and as first- and second-line option in combination with fulvestrant improved progression free survival (PFS) compared to ET alone [5, 6]. Safety analyses showed that diarrhoea was the most common side effect, which could impact adherence to this oral therapy. In these clinical trials, clinician assessment showed approximately 43% of patients experienced clinically significant diarrhoea, usually in early treatment cycles [7]. Treatment discontinuation associated with diarrhoea occurred in fewer than 3% of patients as most managed with dose adjustments or antidiarrheal drugs. Self-reported clinical trial data on quality of life (QoL) showed that global QoL, functioning, pain and most symptoms were consistently maintained from baseline across treatment for patients receiving abemaciclib plus ET [8, 9]. However, diarrhoea was worse in the abemaciclib arm, with the highest symptom burden at earlier visits and return to near baseline levels after treatment.

This information is valuable when prescribing abemaciclib but was obtained from closely monitored clinical trials, so how patients manage side effects in routine clinical care is largely unknown. This is particularly important within the metastatic setting where the trade-off between PFS, side effects and impact on everyday activities, and QoL needs to be considered in treatment decisions [10]. Previous real-world research of CDK4/6 inhibitors has been centred around clinical outcomes [11, 12], and only few studies explored patients’ treatment experiences, including factors affecting adherence, symptom management and QoL [13–16]. Within these reports, data were collected at a single time-point from mixed samples with most patients taking other CDK4/6 inhibitors. A deeper understanding of the symptom burden and self-management of side effects of abemaciclib over a longer period of time is still lacking. Mere reporting of symptoms is insufficient, as the impact of them upon activities of daily living is equally important. The purpose of the current observational mixed-methods study was to explore patients’ experiences in a real-world setting over a period of 6 months, using patient-reported outcome measures and in-depth qualitative interviews in a subset of participants. This paper reports on the incidence and management of the gastrointestinal sideeffects associated with abemaciclib and ET, particularly diarrhoea.

## Methods

### Study sample and procedures

Participants were identified by the clinical teams of 12 UK breast cancer units. Eligible participants were at least 18 years old, female, diagnosed with MBC, scheduled to receive or were receiving abemaciclib in combination with ET (fulvestrant, AIs), able to read English and able to provide informed consent. This study received ethics approval (Tyne & Wear South Research Ethics Committee, 20/NE/0101) and signed informed consent was obtained.

Participants were asked to track bowel movements weekly and to complete QoL questionnaires at study-entry and at 1, 3 and 6 months. Mode of administration (paper or online) was participant’s choice. An optional qualitative interview was offered to all participants and presented elsewhere [17].

### Measures

Participants completed the Diarrhoea Management Diary (DMD) [18], FACT-G [19] with endocrine [20] and diarrhoea [21] subscales, and the Patient Roles and Responsibilities Scale [22]. Only data from the DMD and the FACT diarrhoea subscale are presented, as this paper is focussed on the incidence and management of diarrhoea and other gastrointestinal side effects.

#### The diarrhoea management diary (DMD)

The DMD [18] has eight core closed-ended questions assessing frequency and consistency of bowel movements and use of diarrhoea management strategies, including dietary changes, use of over-the-counter medication, advice from healthcare professionals (HCPs) and treatment adherence (i.e., reducing or discontinuing treatment). Further sub-questions evaluate management and self-care strategies in more detail and probe whether or not they alleviated symptoms. A free-text box was added for patients to provide further insight about their experiences.

#### The FACT diarrhoea subscale

The FACT-G [19] is a well-validated measure of health-related QoL and commonly used within the cancer population. The diarrhoea subscale consists of 11 items relating to gastrointestinal concerns (e.g. *I move my bowels more frequently than usual*) and impact of symptoms (e.g. *I have to limit my social activity because of diarrhoea*) [21]. Responses are based on experiences in the past 7 days, and range from 0 (not at all) to 4 (very much). Items are reverse scored where appropriate and responses summed, giving a possible total score range of 0–44, with higher scores indicating fewer concerns.

#### Demographic and clinical information

Basic demographic information and personal details such as caregiving responsibilities and family situation were collected at study-entry together with details relating to diagnosis and treatment, medication use and comorbidities. Information on treatment interruptions and dose reductions were recorded weekly on the DMD.

### Data analysis

#### The diarrhoea management diary (DMD)

Participants were categorised as having diarrhoea or not at each time-point using the WHO definition of diarrhoea. This definition is used in the European Society for Medical Oncology (ESMO) clinical practice guidelines and is based on both frequency and consistency:—three or more loose/liquid stools per day (frequent passing of formed stools is not diarrhoea) [23, 24]. These categorisations were used to monitor self-reported diarrhoea during the study and to examine the impact of diarrhoea on QoL scores. Descriptive analysis was used for the DMD questions relating to diarrhoea management. Free-text responses were summarised and used to illustrate the findings.

#### The FACT diarrhoea subscale

The minimally important difference (MID) was used to evaluate change from baseline. MID is the smallest change in score that would be perceived by patients to be important, in contrast to a statistically significance difference. These thresholds can be used to determine whether change is of a magnitude likely to be meaningful to the patient [25]. As the diarrhoea subscale does not have an established MID, we used a distribution-based approach to determine meaningful change with the threshold for change set at 0.5 SD change from study-entry [26] resulting in a threshold for meaningful change of ± 5.53 points difference calculated using 0.5SD of the cohort scores at baseline. This threshold was applied to the absolute difference from study-entry to categorise each individual as showing meaningful decline (i.e. a change of 5.53 points or more from their baseline score), no meaningful change, or meaningful improvement from study-entry, at each time-point.

## Results

### Participants

Between September 2020 and November 2022, a total of 57 patients were approached by 10 sites; 46 (81%) of these consented (Online Resource 1). Two women withdrew after study-entry without completing any study measures. Of the remaining 44 participants, 28 completed questionnaires online and 16 on paper.

The demographic and clinical characteristics are summarised in Table 1. Women were aged between 42 and 83 years, and 14/44 (32%) were in paid-employment. Just over half (23/44; 52%) were diagnosed less than a year ago and most had previous treatment. Patients received abemaciclib in combination with fulvestrant (20/44) or AI (24/44). Most (23/44; 52%) completed the baseline measures within 1 week of starting treatment. Prescribed treatment modifications (dose interruptions/reductions) were common; self-reported reasons included side-effects, infections or haematologic toxicities.

**Table 1:** Baseline demographic and clinical patient characteristics (*N* = 44)

**Sociodemographic characteristics**	
*Age in years*	
Mean (SD), median, range	60.2 (9.6), 60, 42–83
*Ethnicity*	
White	40 (91)
Non-White^a^	4 (9)
*Relationship status, n (%)*	
With partner	28 (64)
No partner^b^	16 (36)
*Employment status, n (%)*	
Full-time/part-time work	11 (25)
Self-employed	3 (7)
Unemployed	3 (7)
Long-term sick leave	10 (22.5)
Retired	17 (38.5)
**Clinical characteristics**	
*Time since diagnosis, n (%)*	
< 1 year	23 (52)
1–2 years	1 (2)
> 2 years	20 (46)
*Treatment history, n (%)*	
Surgery	30 (68)
Chemotherapy	31 (71)
Radiotherapy	26 (59)
*Other health condition, n (%)*	22 (50)
*Other medication, n (%)*	22 (50)

^a^Includes African, Asian, Black-Caribbean, Mixed

^b^Includes single, divorced/separated, widowed

### Loss to follow up

Twenty-four (55%) women completed the final study time-point at 6 months, of whom 23 had complete data for all four QoL time-points. Reasons for loss to follow up (Online Resource 1) were balanced between treatment-based (e.g. abemaciclib discontinued, hospitalisation) and study-related or emotional reasons (e.g. stopped responding, being overwhelmed). During the study, treatment was stopped for 10 (23%) women. Reasons for discontinuation included progressive disease (2/10), side effects (4/10; 3 diarrhoea-related), no funding (1/10), allergic reaction (1/10), risk thrombotic event (1/10) and hospitalisation (1/10).

### The diarrhoea management diary

#### Incidence of self-reported diarrhoea

A total of 578 DMDs were completed by 42/44 participants (discrepancies in the numbers are due to incomplete questionnaire data). Thirty-seven (88%) patients completed the DMD at study-entry and at least one subsequent time-point. Nearly half (20/42, 48%) completed the DMD at all 25 time-points. Additional information on bowel movements, stool consistency and self-reported diarrhoea for each time-point is presented in Online Resource 2. Median number of bowel movements varied and was 1–2 stools per day. The frequency ranged from 0 to ≥ 8 stools per day. Stool consistency varied, but was quite soft or hard/firm for most patients. Self-reported diarrhoea peaked earlier in treatment and improved over time. Examples of DMD free-text responses and illustrative quotations (Q) are shown in Table 2 (Online Resource 3 includes a visual representation with additional quotations). Thirty-six patients provided free-text data about diarrhoea experiences, including 16 reporting on other gastrointestinal symptoms, like abdominal pain/cramps, bloating or constipation (Q1, Q2).

**Table 2:** Diarrhoea management diary free-text responses

Context	Free-text responses	Quote	Participant
*Gastrointestinal symptoms*			
GI symptoms	*Two days diarrhoea. Most days bloating, pain and wind*	Q1	No.102, 55y, TP3
GI symptoms	*Out of the last week, five days normal, one day severe constipation, one day severe diarrhoea*	Q2	No.1201, 59y, TP13
Experience of diarrhoea	*Diarrhoea getting worse with excruciating tummy pain and feeling washed out*	Q3	No.502, 63y, TP8
Experience of diarrhoea	*Still no change in bowel habits. Generally, a large soft stool every other day. Diarrhoea usually once a week. Very manageable and no need for loperamide so far*	Q4	No.506, 62y, TP12
No diarrhoea	*I have not had diarrhoea. I have not taken any medication for diarrhoea*	Q5	No.114, 54y, TP1
Change over time	*I have had no diarrhoea this past week which is the first week free on this new medication*	Q6	No.109, 53y, TP7
Change over time	*I seem to have less cramping and two bowel movements. It seems to be easier on me and able to get out a bit now. Still the odd problem day but improving*	Q7	No.510, 76y, TP8
*Management and self-care strategies*			
Dietary changes	*I have had three really bad nights over the last week. After eating risotto, baked potatoes—both would appear to be mild food so it’s hard to make sense of -, one after eating an entire bar of chocolate after having nothing like it for months. I understand that one*	Q8	No.113, 57y, TP7
Dietary changes	*Changed diet to bland diet such as toast, jacket potatoes (not eaten skins), lessen fibre intake—still have diarrhoea*	Q9	No.107, 56y, TP1
Non-prescribed medication	*[I have] more constipation than diarrhoea. Tried prunes also Buscopan for bowel pain/bloating*	Q10	No.102, 55y, TP1
Advice from HCPs	*I spoke to the cancer care nurse from my GP surgery today. She advised cutting out/reducing caffeine and to keep a food diary. I will be trying this, this week*	Q11	No.509, 47y, TP3
*Medication adherence*			
Nonadherence (intentional)	*Was traveling to family—stopped one tablet to cope on journey. Cut out one tablet on return journey*	Q12	No.601, 69y, TP3
Nonadherence (unintentional)	*I went away for a weekend and forgot my pills so missed some doses*	Q13	No.507, 44y, TP12
*Disparity experience and diarrhoea criteria*			
Experience of diarrhoea*	*Sometimes I have no control over my bowel, and it just runs down my legs without realising its coming*	Q14	No.901, 81y, TP3
Impact of diarrhoea***	*This treatment has been worse than the course of chemotherapy I had from [time period]. This treatment being daily, has affected my life and husbands’ life greatly. You cannot plan daily activities, as your symptoms can wipe out any plans you make. Visiting family, planning holidays with family and friends*	Q15	No.601, 69y, TP25
Impact of diarrhoea**	*The diarrhoea was very difficult to manage as there was often no pattern to it, and on several occasions, there was alarmingly little warning. This made me so anxious and nervous about going out. Following one incident whilst out I was too nervous to leave the house for several days*	Q16	No.408, 50y, TP10

*GI* gastrointestinal, *TP* time point, *wk* weeks, *y* age in years

*This participant did not meet the threshold for diarrhoea at any of the 25 time-points completed

**This participant experienced above threshold diarrhoea 5/25 weeks and reported non-adherence 14/25 weeks

***This participant did not meet the threshold to classify as having diarrhoea at any of the 10 time-points completed

**Fig. 1:**
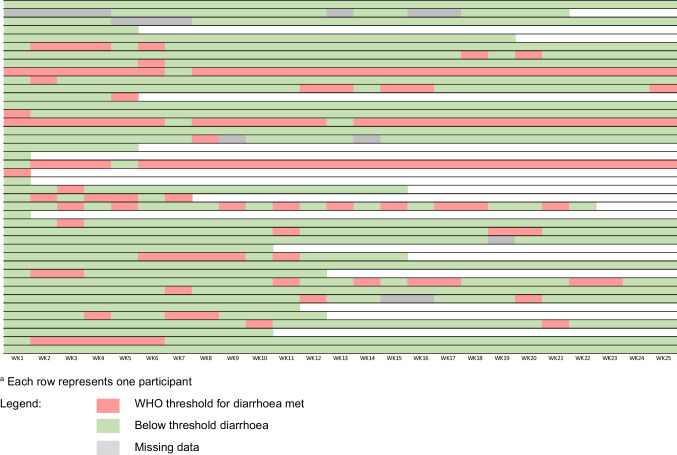
Prevalence of above threshold diarrhoea by participant and week

Participants were classified as reporting diarrhoea or not at each time-point using the WHO criteria of three or more loose/liquid stools per day. Self-reported diarrhoea varied per time-point, affecting between 10 and 24% (Q3, Q4). Most patients were classified as having diarrhoea on a few occasions; however, three had persistent diarrhoea (i.e. 23/24 out of the 25 time-points). Seventeen (40%) patients had no above-threshold diarrhoea while on study (Q5). Figure 1 is a visual representation of the prevalence of diarrhoea for each participant for each study time-point.

#### Diarrhoea management and self-care strategies

Twenty-eight patients (67%) tried one or more management or self-care strategies for diarrhoea, with 21/28 (75%) using one strategy only (Online Resource 4). Interventions were predominantly used within the first 10 weeks. Several patients noted in the free-text responses that diarrhoea reduced during the course of the study (Q6, Q7).

Changing food intake (i.e. avoiding foods or following a diet) was the most frequently used management approach (reported 101 times by 20 patients). This strategy was (a little/quite a bit) successful in most (18/20) patients, although some struggled to find the ‘right’ diet (Q8, Q9).

A third of patients (14/42, 33%) used non-prescribed medication (Q10). This was reported 20 times mainly within the first 9 weeks. Fourteen patients used medication to reduce frequency of bowel movements; 12/14 completed the sub-questions and found this (a little/quite a bit/very) successful. Four tried medication to relieve cramps or abdominal pain, and found this helpful.

Contacting HCPs other than the hospital doctor for advice was only reported on four occasions by four different patients (Q11).

#### Treatment adherence

Diarrhoea was controlled by (non-prescribed) dose adjustments 40 times by ten different patients, of whom six reduced their dose once or twice. Free-text comments showed that self-reported nonadherence was both intentional and unintentional (Q12, Q13). Six patients interrupted their treatment one or more days a week at certain time-points, three of these on more than one occasion.

### The FACT diarrhoea subscale

Total scores on the diarrhoea subscale were wide ranging at each time-point: 3–44 at study-entry (*n* = 42), 4–44 at 1 month (*n* = 38), 4–44 at 3 months (*n* = 29) and 1–44 at 6 months (*n* = 24).

**Fig. 2:**
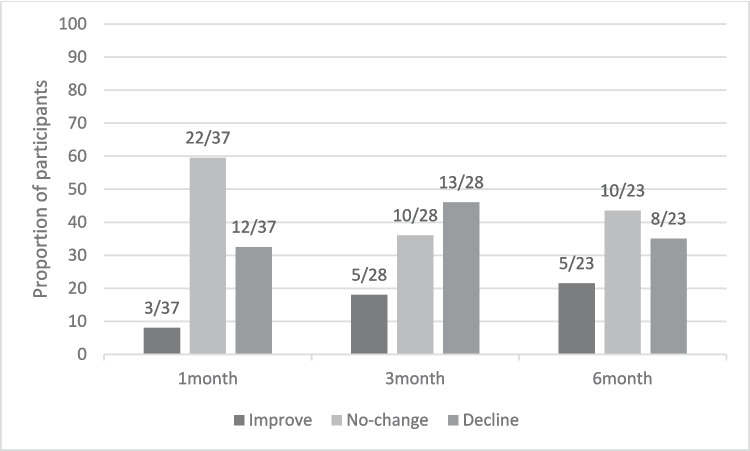
Meaningful change on diarrhoea subscale by time point

Using the MID threshold of ± 5.53, meaningful decline from study-entry in scores on the diarrhoea scale (i.e. increased symptom burden) was reported by 12/37 (32.5%) participants at 1 month, 13/28 (46%) at 3 months and 8/23 (35%) at 6 months.^1^ Figure 2 shows the proportion of participants reporting meaningful improvement (i.e. diarrhoea got better), no change or meaningful decline (i.e. diarrhoea got worse) from study-entry at each time-point.

### Triangulating threshold scores, quality of life and patient experience

#### The diarrhoea subscale and thresholds for diarrhoea

Of those reporting meaningful decline from study-entry on the diarrhoea subscale at each timepoint, only 2/12 were categorised as having diarrhoea at 1 month, 3/13 at 3 months and 2/8 at 6 months. Meaningful decline on the diarrhoea subscale was not associated with reaching threshold for diarrhoea at the corresponding time-point.

Of those categorised as having above threshold diarrhoea, 2/5 also reported meaningful decline on the diarrhoea subscale at 1 month,^2^ 3/4 at 3 months and 2/4 at 6 months.

#### Patient experience and thresholds for diarrhoea

Ten participants reported unplanned (i.e. not discussed with HCP) dose reductions or treatment interruptions to manage diarrhoea. Of these, three completed the study up to 6 months, and only five reached the threshold (≥ 3 loose/liquid stools daily) for diarrhoea on more than one time-point. Free-text data suggest that for some patients, their experience of diarrhoea during study participation did not align with threshold criteria. For example, one woman, who did not meet the threshold for diarrhoea explained having watery stools and loss of bowel control (Q14) leading to a treatment interruption and dose reduction. Other patients who missed the threshold for diarrhoea at some or all time-points described the wide-ranging impact of their experiences on QoL (Q15) and the fear and anxiety associated with this (Q16).

## Discussion

Worldwide, many patients with breast cancer are receiving abemaciclib and ET as first- or second-line treatment for HR + HER2-advanced or metastatic disease, and recently as adjuvant therapy for high-risk early-stage disease [27]. Abemaciclib is taken until disease progression or unacceptable toxicity in MBC. The impact that this oral therapy might have on the quality of patients’ everyday life is therefore significant. To our knowledge, this is the first real-world study collecting longitudinal data on treatment side effects and symptom burden in patients receiving abemaciclib and ET for MBC. The results of this small study showed that treatment-associated diarrhoea can be severe for a limited number of patients, profoundly changing or disrupting their daily activities.

We observed discrepancies between self-reported QoL data, specifically the diarrhoea subscale and formal classification of diarrhoea according to WHO criteria, which is used widely in oncology settings [24]. A significant proportion of our study patients reported scores showing a meaningful decline, despite not having diarrhoea as usually defined. These findings suggest that some of the frequently used thresholds for classifying side effects, such as the WHO and the CTCAE criteria, do not always reflect patients’ experiences [28]. This has been shown in previous studies, including a cross-sectional study in MBC patients taking CDK4/6 inhibitors, where the frequency and severity of treatment-related side effects were consistently greater in patient-reported data than physician-reported data [13, 29–31]. Routinely collecting information about patients’ symptoms and incorporating the outcomes into clinical care, may help ensure that symptoms are adequately captured and managed [32].

The symptom burden of diarrhoea also plays a role in the trade-offs between overall survival and side effects in the treatment of MBC. A recently published UK discrete choice experiment (DCE) study in 105 breast cancer patients about their preferences for MBC treatment, showed that despite a general preference (and underlying belief) in treatment, they are willing to forgo some level of treatment efficacy to avoid particular severity levels of side effects, including diarrhoea. Respondents reported that they would accept a reduction in the probability of overall survival of 6.3% to avoid grade 2 diarrhoea [33]. Similar findings were observed in another DCE study that assessed patients’ preferences for treatments with attributes associated with abemaciclib plus fulvestrant or fulvestrant alone for postmenopausal HR +/HER2- MBC [34]. Though participants had a strong preference for treatment that could extend PFS, even with the risk of grade 2 diarrhoea, when frequency of loose stools increased to grade 3 severity, patients were more willing to sacrifice survival to avoid experiencing more frequent loose stools. These studies suggest that it is important to prevent and manage diarrhoea in patients taking abemaciclib and ET and maintain patients’ motivation for this treatment.

National and international guidelines for the management of diarrhoea associated with cancer treatment predominantly consists of pharmacological management and include educating patients about the potential occurrence of diarrhoea and asking them to proactively administer supportive over-the-counter antidiarrheal medications, usually loperamide, at the first signs of loose stools, increase intake of oral fluids and contact their clinician [24, 35]. Early dose adjustments (omissions or reductions until side effects are easing) are typically recommended when diarrhoea is not resolved and becomes more severe, and have shown to be an effective strategy with no detrimental effect to PFS [36].

Furthermore, a recent survey among 1221 MBC patients about their experiences and perspectives of drug tolerability and willingness to discuss an individualised approach to dosing, showed that the majority (92%) supported this approach [37]. Most (86%) experienced at least one significant treatment-related side effect, including nausea and diarrhoea, that required emergency/hospital care and/or resulted to missing at least one treatment. Respondents believed that frequent patient-physician discussions at the start and throughout treatment may facilitate dose optimization and accommodate patients’ needs and preferences.

In MONARCH 2 and 3, respectively 19% and 14% required dose reductions, and anti-diarrheal drug use was 17% and 61% [5, 38]. A small real-world study in 39 consecutive patients with HR +/HER2- MBC treated with abemaciclib and ET showed that loperamide-based supportive therapy was administered to 72%, and 31% had their dose reduced due to diarrhoea [39]. In our study, half reported that they had treatment modifications and required a scheduled treatment disruption or dose reduction. Use of anti-diarrheal drugs, however, was not systemically monitored, and only self-reported patient data in the free-text responses was used.

Data of nonpharmacological management and self-management of cancer-treatment associated diarrhoea is limited [40]. In our sample, around two-thirds of patients stated that they used self-management strategies to ease diarrhoea or other gastrointestinal symptoms, mainly within the first 3 months of study participation. Very few contacted other HCPs for additional advice. Dietary changes were most frequently reported, by almost one in two patients, followed by use of non-prescribed medication in nearly one in three patients. Most patients either eliminated certain foods from their diet (e.g. fatty foods) or followed a special diet (e.g. low fibre/low residue), with some keeping a food and symptom diary. Others restricted their food intake or had smaller meals, more frequently. The majority reported that this was successful, but often it required a trial-and-error approach.

Only one study to date has examined if food intake impacts on gastrointestinal toxicity in patients with HR +/HER2-MBC receiving abemaciclib monotherapy [41]. Patient-reported data were captured daily via e-diaries which facilitated detailed, real-time description of the patient experience. The results suggested that diarrhoea incidence was unrelated to timing of food intake, but did not address food composition intake, which needs further investigation.

It is well-known that, generally, lack of adherence to recommended therapy will reduce the efficacy of treatment. In this study, medication nonadherence was disclosed by ten patients to help to control their bowel movements, with three using this self-management strategy frequently. Two prior studies investigated potential challenges and barriers with adherence or persistence to CDK4/6 inhibitors in MBC patients. Stephenson et al. [14] reported few adherence issues in a small sample of patients and stated that a trusted relationship with HCPs, positive views and belief in the importance of their medication, strong medical and social support and minimal personal drug costs were important factors for optimal adherence. In contrast, qualitative research by Conley et al. [15] found that barriers to adherence to CDK4/6 inhibitors existed on multiple levels. Treatment side effects, costs, patients’ knowledge about the prescribed medication and communication with their care team were related to adherence behaviour. The authors suggested that assessing these factors at the start of treatment and periodically thereafter could potentially identify issues earlier and address them sooner. A large randomised trial, examining if a new strategy for continuous support, an eHealth-platform, can help therapy management and HCP-patient interaction in those taking CDK4/6 inhibitors, is currently underway [42].

There are limitations to this explorative longitudinal study. Recruitment was low, and almost half of patients were lost to follow up. However, this is not uncommon in metastatic cancer settings [43, 44] and was further complicated by conducting a clinical study during a global pandemic when many experienced multiple interruptions (e.g. temporary recruitment pauses with restarts, delayed enrolment) [45]. There are also challenges to off-site recruitment into real-world trials, including for the current study. Patients often commenced treatment directly following the clinician consultation and before consent could be taken. Lastly, a large survey showed that the risk of diarrhoea was one of the key drivers of both oncologist and patient preferences for CDK4/6 inhibitor treatment choice [46]. Overall, palbociclib plus AI was most preferred, which could explain lower prescription rates for abemaciclib.

In conclusion, treatment with abemaciclib and ET is associated with profound diarrhoea in a number of patients with considerable consequences for everyday life. More scientific enquiry into the reasons why some patients appear to be resistant to the development of gastrointestinal side effects might be interesting. A more holistic approach to monitoring diarrhoea, involving discussions around impact on patients’ QoL, including fear of its incidence alongside prospective guidance on diarrhoea management is needed. This would better support these patients tolerate the treatment they often highly value and could reduce nonadherence. Further research into communication skills training could support HCPs in discussing treatment benefits, side effects and adherence. Such a programme could be developed with multi-disciplinary teams and patient representatives and evaluated in both metastatic and adjuvant breast cancer treatment settings.

## Supplementary Information

Below is the link to the electronic supplementary material.

Supplementary file1 (DOCX 615 KB)

Supplementary file2 (DOCX 31 KB)

Supplementary file3 (DOCX 311 KB)

Supplementary file4 (DOCX 29 KB)

## Data Availability

De-identifed data may be made available upon request only.

## Footnotes

1 One participant did not complete the diarrhoea subscale at baseline so it was not possible to calculate meaningful change.

2 6 people had above threshold diarrhoea at one month but only five had diarrhoea subscale data.
