# Supplementary material for: Incidence and management of diarrhoea associated with abemaciclib and endocrine therapy for hormone-receptor positive, HER2-negative metastatic breast cancer: the UK patients’ experiences
Source: Support Care Cancer. 2025 Apr 26;33(5):422. doi: 10.1007/s00520-025-09440-7 (PMC12033210; doi:10.1007/s00520-025-09440-7)
Supplement: Supplementary file 2 — Supplementary file2 (DOCX 31 KB) [file 520_2025_9440_MOESM2_ESM.docx]

**Online Resource 2**

**Journal**

Supportive Care in Cancer

**Title**

Incidence and management of diarrhoea associated with abemaciclib and endocrine therapy for hormone-receptor positive, HER2 negative metastatic breast cancer: the UK patients’ perspectives

**Authors**

Helena Harder^1^ (0000-0002-7296-8227)

Rachel Starkings^1^ (0000-0002-1947-018X)

Lesley Fallowfield^1^ (0000-0003-0577-4518)

Shirley May^1^ (0000-0002-3167-9891)

Valerie Shilling^1^ (0000-0002-5610-0321)

**Affiliations**

^1^ Sussex Health Outcomes Research and Education in Cancer (SHORE-C), Brighton and Sussex Medical School, University of Sussex, Brighton, United Kingdom

**Corresponding author**

Dr Helena Harder

Sussex Health Outcomes Research and Education in Cancer (SHORE-C)

Brighton and Sussex Medical School, University of Sussex, Brighton, UK

[h.harder@sussex.ac.uk](mailto:h.harder@sussex.ac.uk)

**Table I - Bowel movements, stool consistency and self-reported diarrhoea on DMD**

| Follow-up in weeks^a^ | Wk1, n=42 | Wk2, n=37 | Wk3, n=37 | Wk4, n=37 | Wk5, n=37 |
| --- | --- | --- | --- | --- | --- |
| Stools per day, median  stools per day, range | 1  0-6 | 2  0-≥8 | 2  0-≥8 | 2  0-5 | 2  0-7 |
| Hard/firm, %  Quite soft, %  Very soft/loose, %  Watery, % | 34.1*  43.9*  17.1*  4.9* | 27.0  32.4  29.7  10.8 | 24.3  35.1  27.0  13.5 | 16.7**  47.2**  27.8**  8.3** | 24.3  43.2  27.0  5.4 |
| Self-reported diarrhoea^b^ | 4 (10%)* | 8 (22%) | 9 (24%) | 7 (19%)** | 6 (16%) |
| Diarrhoea ≥5 days that week^c^ | 3/4 | 6/8 | 6/9 | 2/7 | 5/6 |
| Follow-up in weeks | **Wk6, n=34** | **Wk7, n=34** | **Wk8, n=34** | **Wk9, n=33** | **Wk10, n=34** |
| Stools per day, median  Stools per day, range | 2  1-≥8 | 2  0-6 | 2  0-6 | 2  0-5 | 2  0-6 |
| Hard/firm, %  Quite soft, %  Very soft/loose, %  Watery, % | 20.6  44.1  26.5  8.8 | 29.4  29.4  32.4  8.8 | 32.4  32.4  29.4  5.9 | 24.2  51.5  18.2  6.1 | 29.4  47.1  17.6  5.9 |
| Self-reported diarrhoea | 7 (21%) | 5 (15%) | 6 (18%) | 5 (15%) | 4 (12%) |
| Diarrhoea ≥5 days that week^c^ | 3/7 | 3/5 | 2/6 | 4/5 | 3/4 |
| Follow-up in weeks | **Wk11, n=32** | **Wk12, n=31** | **Wk13, n=28** | **Wk14, n=28** | **Wk15, n=28** |
| Stools per day, median  Stools per day, range | 2  0-≥8 | 2  0-5 | 2  0-5 | 2  0-6 | 1.5  0-5 |
| Hard/firm, %  Quite soft, %  Very soft/loose, %  Watery, % | 21.9  46.9  28.1  3.1 | 32.3  48.4  16.1  3.2 | 14.3  60.7  17.9  7.1 | 25.0  50.0  21.4  3.6 | 28.6  39.3  28.6  3.6 |
| Self-reported diarrhoea | 7 (22%) | 5 (16%) | 4 (14%) | 4 (14%) | 5 (18%) |
| Diarrhoea ≥5 days that week^c^ | 5/7 | 4/5 | 2/4 | 3/4 | 4/5 |
| Follow-up in weeks | **Wk16, n=25** | **Wk17, n=26** | **Wk18, n=27** | **Wk19, n=26** | **Wk20, n=26** |
| Stools per day, median  Stools per day, range | 1  0-5 | 1.5  0-6 | 1  0-5 | 1  0-7 | 2  0-6 |
| Hard/firm, %  Quite soft, %  Very soft/loose, %  Watery, % | 12.0  56.0  24.0  8.0 | 30.8  46.2  19.2  3.8 | 25.9  51.9  18.5  3.7 | 30.8  42.3  19.2  7.7 | 30.8  34.6  26.9  7.7 |
| Self-reported diarrhoea | 5 (20%) | 5 (19%) | 5 (18.5%) | 4 (15%) | 6 (23%) |
| Diarrhoea ≥5 days that week^c^ | 3/5 | 3/5 | 3/5 | 3/4 | 2/6 |
| Follow-up in weeks | **Wk21, n=26** | **Wk22, n=25** | **Wk23, n=24** | **Wk24, n=24** | **Wk25, n=24** |
| Stools per day, median  Stools per day, range | 1.5  0-6 | 1  0-7 | 1.5  0-6 | 1  0-6 | 1  0-≥8 |
| Hard/firm, %  Quite soft, %  Very soft/loose, %  Watery, % | 26.9  53.8  15.4  3.8 | 12.0  72.0  12.0  4.0 | 29.2  45.8  20.8  4.2 | 33.3  50.0  12.5  4.2 | 25.0  54.2  12.5  8.3 |
| Self-reported diarrhoea | 5 (19%) | 4 (16%) | 4 (17%) | 3 (12.5%) | 4 (17%) |
| Diarrhoea ≥5 days that week^c^ | 3/5 | 2/4 | 2/4 | 2/3 | 3/4 |

Abbreviations: Wk = week; DMD = Diarrhoea Management Diary [1]

^a^ Numbers at follow-up do not equal 43 due to study attrition and non/incomplete response

^b^ Using the WHO definition of three or more loose or liquid stools

^c^ Number of patients experiencing diarrhoea, who selected ‘every day’ or ‘almost every day (5-6 days) on DMD item 2 (‘Over the past week how many days were typically like this?’)

**n=41; **n=36*

1. *Harder, H., et al., The development and initial evaluation of the Diarrhoea Management Diary (DMD) in patients with metastatic breast cancer. Breast Cancer Research and Treatment, 2020. 183(3): p. 629-638*
