# Supplementary material for: Incidence and management of diarrhoea associated with abemaciclib and endocrine therapy for hormone-receptor positive, HER2-negative metastatic breast cancer: the UK patients’ experiences
Source: Support Care Cancer. 2025 Apr 26;33(5):422. doi: 10.1007/s00520-025-09440-7 (PMC12033210; doi:10.1007/s00520-025-09440-7)

**Online Resource 3**

**Journal**

Supportive Care in Cancer

**Title**

Incidence and management of diarrhoea associated with abemaciclib and endocrine therapy for hormone-receptor positive, HER2 negative metastatic breast cancer: the UK patients’ perspectives

**Authors**

Helena Harder^1^ (0000-0002-7296-8227)

Rachel Starkings^1^ (0000-0002-1947-018X)

Lesley Fallowfield^1^ (0000-0003-0577-4518)

Shirley May^1^ (0000-0002-3167-9891)

Valerie Shilling^1^ (0000-0002-5610-0321)

**Affiliations**

^1^ Sussex Health Outcomes Research and Education in Cancer (SHORE-C), Brighton and Sussex Medical School, University of Sussex, Brighton, United Kingdom

**Corresponding author**

Dr Helena Harder

Sussex Health Outcomes Research and Education in Cancer (SHORE-C)

Brighton and Sussex Medical School, University of Sussex, Brighton, UK

[h.harder@sussex.ac.uk](mailto:h.harder@sussex.ac.uk)


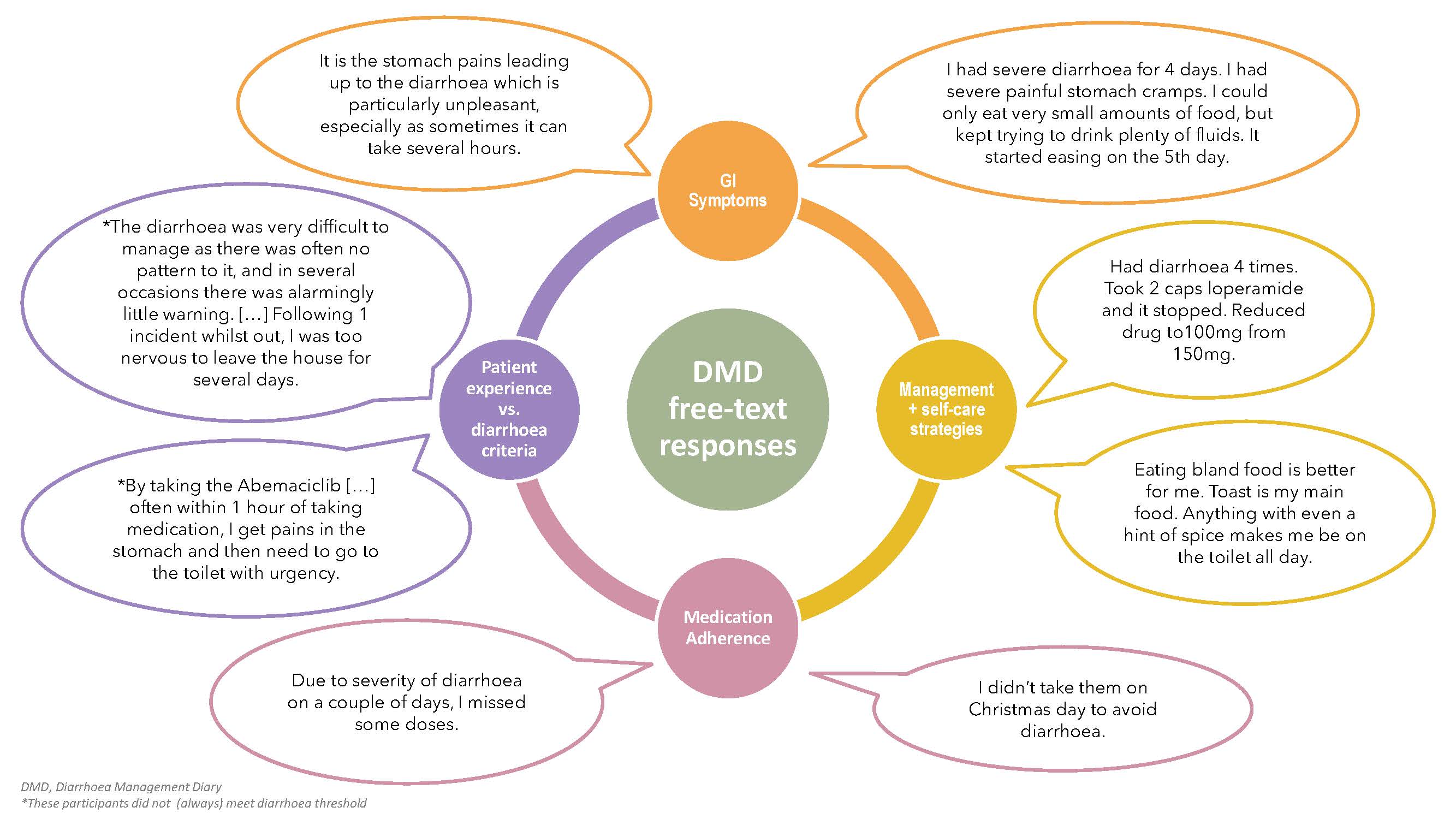

Supplement: Supplementary file 3 — Supplementary file3 (DOCX 311 KB) [file 520_2025_9440_MOESM3_ESM.docx]
