# Supplementary material for: Incidence and management of diarrhoea associated with abemaciclib and endocrine therapy for hormone-receptor positive, HER2-negative metastatic breast cancer: the UK patients’ experiences
Source: Support Care Cancer. 2025 Apr 26;33(5):422. doi: 10.1007/s00520-025-09440-7 (PMC12033210; doi:10.1007/s00520-025-09440-7)
Supplement: Supplementary file 4 — Supplementary file4 (DOCX 29 KB) [file 520_2025_9440_MOESM4_ESM.docx]

**Online Resource 4**

**Journal**

Supportive Care in Cancer

**Title**

Incidence and management of diarrhoea associated with abemaciclib and endocrine therapy for hormone-receptor positive, HER2 negative metastatic breast cancer: the UK patients’ perspectives

**Authors**

Helena Harder^1^ (0000-0002-7296-8227)

Rachel Starkings^1^ (0000-0002-1947-018X)

Lesley Fallowfield^1^ (0000-0003-0577-4518)

Shirley May^1^ (0000-0002-3167-9891)

Valerie Shilling^1^ (0000-0002-5610-0321)

**Affiliations**

^1^ Sussex Health Outcomes Research and Education in Cancer (SHORE-C), Brighton and Sussex Medical School, University of Sussex, Brighton, United Kingdom

**Corresponding author**

Dr Helena Harder

Sussex Health Outcomes Research and Education in Cancer (SHORE-C)

Brighton and Sussex Medical School, University of Sussex, Brighton, UK

[h.harder@sussex.ac.uk](mailto:h.harder@sussex.ac.uk)

**Table II - Diarrhoea management and self-care strategies measured on the DMD**

| Follow-up in weeks^a^ | Wk1, n=41 | Wk2, n=37 | Wk3, n=37 | Wk4, n=37 | Wk5, n=37 |
| --- | --- | --- | --- | --- | --- |
| Dietary changes^b^ | 7 (17%) | 7 (19%) | 12 (32%) | 11 (30%) | 8 (22%) |
| Use of non-prescribed drugs^c^ | 3 (7%) | 4 (11%) | 5 (14%) | 2 (5%) | 1 (3%) |
| Contact/advice from HCP^d^ | 0 | 0 | 2 (5%) | 0 | 1 (3%) |
| Reducing oral treatment^e^ | 1/27 | 1/31 | 2/31 | 2/32 | 3/32 |
| Discontinuing oral treatment^e^ | 1/29 | 1/32 | 1/32 | 2/33 | 3/33 |
| Follow-up in weeks | **Wk6, n=34** | **Wk7, n=34** | **Wk8, n=34** | **Wk9, n=33** | **Wk10, n=34** |
| Dietary changes^b^ | 3 (9%) | 6 (18%) | 6 (18%) | 4 (12%) | 6 (18%) |
| Use of non-prescribed drugs^c^ | 1 (3%) | 0 | 1 (3%) | 2 (6%) | 0 |
| Contact/advice from HCP^d^ | 0 | 0 | 0 | 0 | 0 |
| Reducing oral treatment^e^ | 1/31 | 3/30 | 5/31 | 1/30 | 3/32 |
| Discontinuing oral treatment^e^ | 1/31 | 1/31 | 4/31 | 1/31 | 1/32 |
| Follow-up in weeks | **Wk11, n=32** | **Wk12, n=31** | **Wk13, n=28** | **Wk14, n=28** | **Wk15, n=28** |
| Dietary changes^b^ | 3 (9%) | 2 (7%) | 3 (11%) | 3 (11%) | 2 (7%) |
| Use of non-prescribed drugs^c^ | 0 | 0 | 0 | 0 | 0 |
| Contact/advice from HCP^d^ | 0 | 0 | 0 | 0 | 0 |
| Reducing oral treatment^e^ | 3/30 | 2/27 | 2/27 | 3/27 | 1/28 |
| Discontinuing oral treatment^e^ | 0 | 0 | 1/27 | 2/28 | 1/28 |
| Follow-up in weeks | **Wk16, n=25** | **Wk17, n=26** | **Wk18, n=27** | **Wk19, n=26** | **Wk20, n=26** |
| Dietary changes^b^ | 2 (8%) | 2 (8%) | 1 (4%) | 2 (8%) | 4 (15%) |
| Use of non-prescribed drugs^c^ | 0 | 0 | 0 | 0 | 1 (4%) |
| Contact/advice from HCP^d^ | 0 | 0 | 0 | 0 | 1 (4%) |
| Reducing oral treatment^e^ | 0 | 0 | 0 | 1/25 | 2/25 |
| Discontinuing oral treatment^e^ | 0 | 0 | 0 | 1/25 | 0/25 |
| Follow-up in weeks | **Wk21, n=26** | **Wk22, n=25** | **Wk23, n=24** | **Wk24, n=24** | **Wk25, n=24** |
| Dietary changes^b^ | 1 (4%) | 2 (8%) | 1 (4%) | 2 (8%) | 1 (4%) |
| Use of non-prescribed drugs^c^ | 0 | 0 | 0 | 0 | 0 |
| Contact/advice from HCP^d^ | 0 | 0 | 0 | 0 | 0 |
| Reducing oral treatment^e^ | 0 | 1/24 | 2/22 | 1/22 | 1/21 |
| Discontinuing oral treatment^e^ | 0 | 1/25 | 2/22 | 1/21 | 1/20 |

Abbreviations: Wk = week; DMD = Diarrhoea Management Diary [1]

^a^ Numbers at follow-up do not equal 43 due to study attrition and non/incomplete response

^b^ Avoiding certain foods and/or following a special diet

^c^ Use of medication not prescribed by hospital doctor (i.e. drugs to reduce bowel frequency or cramping/pain)

^d^ Other than hospital doctor (e.g. general practitioner, pharmacist)

^e^ Numbers only; may include patients who had a prescribed treatment break or dose reduction that week

1. *Harder, H., et al., The development and initial evaluation of the Diarrhoea Management Diary (DMD) in patients with metastatic breast cancer. Breast Cancer Research and Treatment, 2020. 183(3): p. 629-638*
